# Physical Characteristics of Tetrahydroxy and Acylated Derivatives of Jojoba Liquid Wax in Lubricant Applications

**DOI:** 10.1155/2018/7548327

**Published:** 2018-02-01

**Authors:** Rogers E. Harry-O'kuru, Girma Biresaw, Sherald Gordon, Jingyuan Xu

**Affiliations:** ^1^Bio-Oils Research Unit, United States Department of Agriculture-Agricultural Research Service, 1815 North Street, Peoria, IL 61604, USA; ^2^Plant Polymer Research Unit, National Center for Agricultural Utilization Research, United States Department of Agriculture-Agricultural Research Service, 1815 North Street, Peoria, IL 61604, USA

## Abstract

Jojoba liquid wax is a mixture of esters of long-chain fatty acids and fatty alcohols mainly C38:2–C46:2. The oil exhibits excellent emolliency on the skin and, therefore, is a component in many personal care cosmetic formulations. The virgin oil is a component of the seed of the jojoba (*Simmondsia chinensis*) plant which occurs naturally in the Sonora Desert in the United States and northwestern Mexico as well as in the northeastern Sahara desert. The seed contains 50–60% oil by dry weight. The plant has been introduced into Australia, Argentina, and Israel for commercial production of the jojoba oil. As a natural lubricant, we are seeking to explore its potential as a renewable industrial lubricant additive. Thus, we have chemically modified the carbon-carbon double bonds in the oil structure in order to improve its already good resistance to air oxidation so as to enhance its utility as well as its shelf life in nonpersonal care applications. To achieve this goal, we have hydroxylated its –C=C– bonds. Acylation of the resulting hydroxyl moieties has generated short-chain vicinal acyl substituents on the oil which keep the wax liquid, improving its cold flow properties and also protecting it from auto-oxidation and rancidity.

## 1. Introduction

The jojoba plant, *Simmondsia chinensis* (Link), is native to the Sonora Desert of northwestern Mexico, California and neighboring states [1]. It produces capsules with seeds which are rich in a unique oil that is actually a liquid wax. At the molecular unit, jojoba oil unlike most vegetable oils is not a triglyceride, that is, fatty acids esterified to a glycerol backbone. Rather, each molecule of this oil comprises a linear long-chain monoene fatty acid esterified to a long-chain monoene fatty alcohol giving rise to mainly C38:2–C46:2 liquid wax esters. The oil content of the seed is 50–60% by weight of dry matter [2–8]. Jojoba oil is highly emollient and lends itself to use as a component in myriads of personal care cosmetic formulations. Derivatization of this oil has been practiced for many years producing halogenated products from which polyunsaturated analogues of jojoba oil intermediates were generated for research and other needs [9–13]. Many more potential uses of the oil have been anticipated including biodiesel manufacture [14], but suitability of this oil for use as a fuel is questionable technically unless it is cracked to shorter chain-length intermediates. Because of its natural lubricating character, it has been explored as an anticorrosive agent for iron [15]. Although jojoba oil has enormous use potential, its drawback is its expensiveness arising from the limited commercial volume of oil available. To offset this, handicap plantations for commercial production of jojoba oil are developing in Argentina, Australia, Israel, and elsewhere on semiarid lands [12]. Recent efforts to explore synthetic and enzymatic development of jojoba-like mimics or analogue esters are yet other approaches aimed at narrowing the availability gap [16, 17]. The latter approaches will become more practical when long-chain fatty acids from oilseed crops are commercially available using developing improved separation techniques for such oils. The ready availability of these long-chain fatty acids and fatty alcohols as synthons will greatly facilitate synthetic approaches to jojoba-like esters. In the current study, the aim is to tap the natural emolliency of the oil into an improved cold flow-modified jojoba oil in the form of the acetate, propionate, and valerate esters that act as lubricant additive agents for base lubricant oils.

## 2. Materials and Methods

### 2.1. Materials

Jojoba oil was donated by Purcell Jojoba International (Avila Beach, CA); formic acid, 90.4% and hydrogen peroxide (50%), acetic anhydride, propionic and valeric anhydrides were purchased from Sigma-Aldrich (St. Louis MO); whereas MgSO_4_, Na_2_CO_3_, NaCl, HCl, NaHCO_3_, and *p*-TsOH were from Thermo Fisher (Chicago, IL). High-oleic sunflower oil, with 81% oleic acid, was obtained from Columbus Foods Company (Des Plaines, IL). Polyalpha olefin of viscosity 6 cSt at 100°C (PAO-6) supplied under the trade name Durasyn 166 by Ineos Oligomers (League City, TX) was a free sample. All reagents and solvents were used as supplied. Steel balls used in 4-ball experiments were obtained from Falex Corporation (Aurora, IL) and have the following specifications: material, chrome-steel alloy made from AISI E52100 standard steel; hardness, 64–66 *R*_*c*_; diameter, 12.7 mm; and finish, grade 25 extra polish. The steel balls were cleaned prior to use in 4-ball experiments by consecutive 10 min sonications in isopropyl alcohol and hexane solvents.

### 2.2. Methods

#### 2.2.1. Instrumentation

- Fourier-transform infrared (FT-IR) spectrometry: FT-IR spectra were measured on an arid zone FT-IR spectrometer (ABB MB-Series, Houston, TX, USA) equipped with a DTGS detector. Liquid derivatives were pressed between two NaCl discs (25 mm × 5 mm) to give thin transparent oil films for analysis by FT-IR spectrometry. Absorbance spectra were acquired at 4 cm^−1^ resolution and signal-averaged over 32 scans. Interferograms were Fourier transformed using cosine apodization for optimum linear response. Spectra were baseline corrected, scaled for mass differences, and normalized to the methylene peak at 2927 cm^−1^.
- Nuclear magnetic resonance spectroscopy: ^1^H and ^13^C NMR spectra were acquired on a Bruker AV-500 MHz spectrometer with a dual 5 mm proton/carbon probe (Bruker, Ballerica, MA, USA). The internal standard used was tetramethylsilane.
- Density, viscosity, and viscosity index (VI): density and dynamic and kinematic viscosities at 40, 75, and 100°C were measured on a Stabinger SVM3000/G2 viscometer (Anton Paar GmbH, Graz, Austria) according to ASTM D-7042 [18]. VI was calculated from kinematic viscosity data at 40 and 100°C according to ASTM D 2270-93 [19].
- Solubility: solubility (% w/w) of jojoba and derivatives at room temperature in polyalpha olefin (PAO-6) base oils was determined gravimetrically, by visual inspection for any changes in oil transparency with increased solute concentration.
- Pressurized differential scanning calorimetry (PDSC): PDSC tests were conducted on a Q20P pressure differential scanning calorimeter (TA Instruments-Waters LLC, New Castle, DE) according to ASTM D-6186 [20]. Details of the test procedure have been given before [21]. The instrument is fitted with a computer and appropriate software to allow for data acquisition and analysis. Tests were conducted with the cell pressurized with pure oxygen to 500 ± 25 psig in the dynamic mode, that is, with a positive oxygen flow rate of 100 ± 10 mL/min. Duplicate runs were conducted, and average values of onset temperature (OT) and peak temperatures (PTs) are reported.
- Four-ball (4-ball) tribological test instrument: tests were conducted on a model KTR-30L 4-ball tribometer (Koehler Instrument Company, Bohemia, NY), equipped with TriboDATA software (Koehler Instrument Company, Bohemia, NY). Details of the instrument hardware and software can be found elsewhere [22].
- Four-ball antiwear (AW) tests: antiwear tests were conducted according to ASTM D 4172-94 [23]. The coefficient of friction (COF) from each test was calculated from the corresponding torque and load data in accordance with the ASTM D 5183 procedure [24]. After each test, the wear scar diameters along and across the wear direction of the three bottom balls were measured using a wear scar measurement system comprising hardware and ScarView software (Koehler Instrument Company, Inc., Bohemia, NY). Each test lubricant was used in at least two AW measurements, and average COF and wear scar diameter (WSD) values are reported.
- Four-ball extreme pressure (EP) tests: extreme pressure tests were conducted according to ASTM D 2783 [25]. The method involves a series of 10-second tests, conducted with progressively increasing load, until welding of the four balls is observed. The load at which welding occurs is reported as the weld point of the lubricant tested. Weld point is an inherent property of lubricants and higher weld point corresponds to superior EP property.

### 2.3. Data Analysis

Data analysis was conducted using IgorPro version 5.0.3.0 software (WaveMetrics, Inc., Lake Oswego, OR).

#### 2.3.1. Synthesis of Tetrahydroxy Jojoba Oil

**Figure 1:**
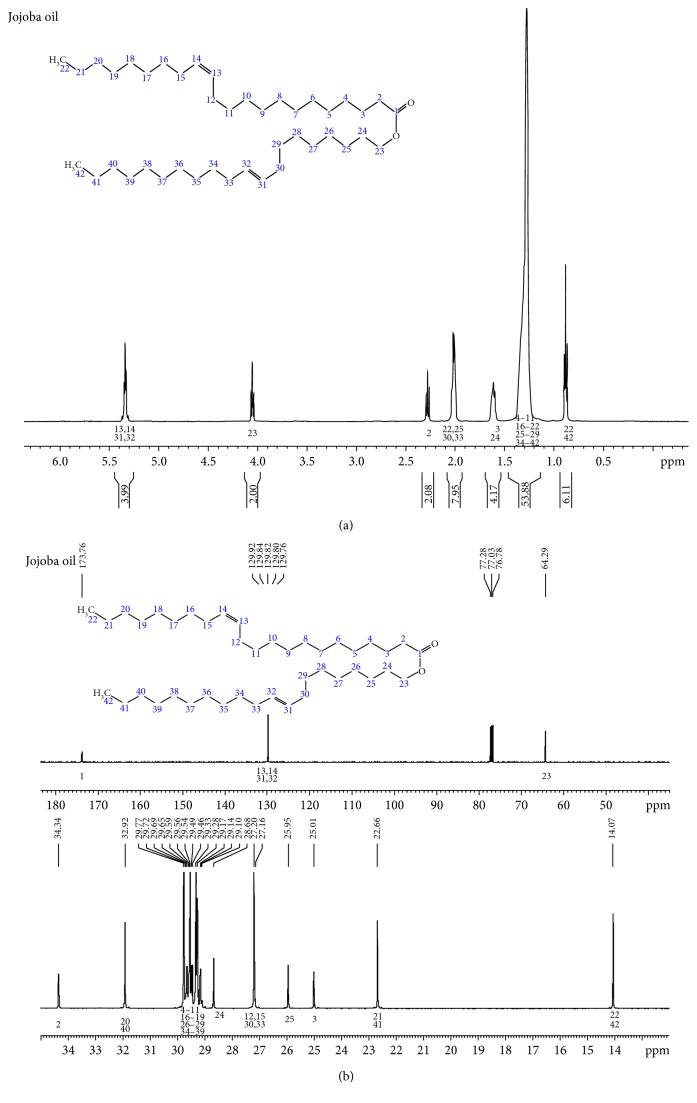
(a) ^1^H NMR spectrum of unmodified jojoba oil. (b) ^13^C NMR spectrum of unmodified jojoba oil.

Jojoba oil (241.5 g, 391.7 mmol) based on the C42 content was placed in a dry 1.0 liter jacketed reaction vessel equipped with an overhead stirrer. The oil was stirred vigorously at 40°C, and formic acid (36.6 g, 795 mmol) was added in one portion. Hydrogen peroxide (50%, 80 g, 3.35 mol) was then added dropwise to the reaction mixture. At the end of peroxide addition, the temperature was raised to 70°C, and the progress of the reaction was monitored at 30 min intervals by FT-IR spectrometry of withdrawn samples. Progress in attenuation of the 3010–3006 cm^−1^ band of the olefin with time and increasing formation of the oxirane band around 840 cm^−1^ was monitored in 30 min intervals of the reaction. The reaction was judged complete in about 3 h. Heating was then discontinued, allowing the mixture to cool to near room temperature whence it was transferred into a separatory funnel for phase separation. The aqueous layer was removed and discarded. An aliquot of the organic phase (oxirane) was removed for analysis (Figure 1), whereas the bulk of the organic layer was returned to the reaction flask to which 300 mL of 6 M HCl was added. This mixture was stirred and heated to 70°C to ring-open the oxirane. At disappearance of the 847 cm^−1^ band, heating was discontinued and the mixture cooled and transferred into a beaker of saturated NaHCO_3_ solution. After cessation of effervescence, the mixture was poured into a separatory funnel and the layers were allowed to separate. The aqueous layer was removed and extracted with 2 × 100 mL of ethyl acetate. The acetate extract was combined with the organic phase and washed with brine, and the organic layer was dried over MgSO_4_. The dried organic layer was filtered, and the solvent was removed under reduced pressure by rotary evaporation to give 264.3 g (98.5%) of the tetrahydroxy jojoba oil. FT-IR *ν*_NaCl_ cm^−1^: 3454, 2925, 2855, 1736, 1462, 1376, 1242, 1176, 1043, 722. ^1^H NMR (CDCl_3_) *δ*: 4.01 *t* (*J* = 6.8, 6.7 Hz, 2H), 3.85 m (2H), 3.58 m (2H), 2.25 *t* (*J* = 7.5, 7.5 Hz, 2H), 2.02 s, 1.75 m (4H), 1.55 m (5H), 1.5 m (5H), 1.4 m (5H), 1.25 bs (51H), 0.85 *t* (*J* = 6.6, 6.7 Hz, 6H); ^13^C (CDCl_3_) *δ*: 173.97, 74.42, 73.84, 68.77, 68.76, 64.37, 34.76, 34.45, 34.32, 33.53, 31.85, 31.83, 31.81, 29.69, 29.66, 29.54, 29.51, 29.49, 29.43, 29.40, 29.34, 29.30, 29.26, 29.22, 29.19, 29.12, 29.07, 28.60, 26.68, 25.89, 25.67, 25.64, 24.95, 22.61, 20.58, 14.05. The unmodified jojoba oil shows ^1^H NMR (CDCl_3_) *δ*: 5.48 m (4H), 4.08 *t* (*J* = 7.4, 6.7 Hz, 2H), 2.3 *t* (*J* = 8.2, 7.5 Hz, 2H), 2.05 m (8H), 1.64 m (4H), 1.3 bs (55H), 0.90 *t* (*J* =  6.6, 7 Hz, 6H); ^13^C NMR *δ*: 173.97, 129.89, 129.87, 128.85, 129.81, 64.35, 34.39, 31.91, 29.77, 29.71, 29.69, 29.65, 2957, 2953, 29.49, 29.45, 29.33, 29.30, 29.28, 29.17, 28.67, 27.21, 25.95, 25.02, 22.68, 14.09. FT-IR of oxirane intermediate *ν*_NaCl_ cm^−1^: 2985, 2956, 2919, 2874, 2852, 1735, 1476, 1380, 1241, 1209, 1185, 847, 721.

#### 2.3.2. Acetylation of Tetrahydroxy Jojoba Oil

Tetrahydroxy jojoba oil (178.7 g, 251.4 mmol), acetic anhydride (216.0 g, 2.12 mol), and p-toluene sulfonic acid (1.0 g) were placed into a flame-dried 1.0 liter round-bottomed flask (RBF) fitted with a heating mantle, a magnetic stir bar, and a reflux condenser protected with a drierite tube. The reaction mixture was stirred and heated to gentle reflux overnight. The reaction was stopped when FT-IR spectrum of a worked up sample of the reaction mixture showed no (OH) absorbance. The product mixture was cooled, diluted with ethyl acetate, and poured into stirring saturated NaHCO_3_ solution. More solid NaHCO_3_ was added until no more effervescence occurred. The mixture was then poured into a separatory funnel and partitioned. The aqueous layer was then re-extracted with ethyl acetate (2 × 100 mL); this extract was combined with the organic phase, washed with brine, and dried over MgSO_4_. The dried material was filtered and concentrated under reduced pressure to give 188.7 g (98%), d23°C 1.031 g/mL; *ν*_NaCl_: 2927 vs, 2857 s, 1743 vs, 1464 m, 1372 m-s, 1232 vs, 1180 m, 1022 m, 725 m. 1H NMR (CDCl3) *δ*: 5.05 m (2H), 4.04 *t* (*J* = 6.7 Hz, 2H), 3.90 m (2H), 2.25 *t* (*J* = 7.5 Hz, 2H), 2.05 s, (6H), 1.65 m (12H), 1.5 m (3H), 1.25 m (57H), 0.85 *t* (*J* = 6.6, 7.1 Hz, 7H). 13C NMR (CDCl3) *δ*: 173.79, 170.44, 170.40, 75.05, 73.81, 64.29, 63.62, 34.30, 34.27, 31.78, 31.14, 30.69, 29.65, 29.58, 29.56, 29.52, 29.47, 29.41, 29.38, 29.34, 29.32, 29.27, 29.20, 29.14, 29.07, 29.01, 28.97, 28.62, 26.52, 25.89, 25.27, 25.10, 24.94, 22.59, 20.86, 14.04.

#### 2.3.3. Propionate Ester of Tetrahydroxy Jojoba Oil

Tetrahydroxy jojoba oil (49.8 g, 72.5 mmol) was placed in a flame-dried 250 mL RBF containing a magnetic stir bar and *p-*TsOH (0.5 g) as a catalyst. To the mixture, propionic anhydride (97%, 100 mL, 101 g, 0.78 mol) was added in one portion. A water-cooled reflux condenser with a drying tube was attached, and the reaction mixture was stirred and gently heated overnight as in the acetate above. When an assayed sample of the reaction mixture showed no OH absorbance in its FT-IR spectrum, propanoylation of the starting substrate was judged complete and the heat source was removed. The reaction mixture was allowed to cool to near room temperature whence it was poured into saturated NaHCO_3_ solution. More bicarbonate was added into the workup mixture until no effervescence was observed. The mixture was then transferred into a separatory funnel, and the partitioned layers were separated. The aqueous phase was extracted with EtOAc (100 mL × 2), and the extract was combined with the organic phase. The latter phase was washed twice with brine, dried over MgSO_4_, and concentrated to give 192 g, 86.9% yield; d^40^ 0.9618 g·cm^3^. FT-IR spectrum *ν*_NaCl_ cm^−1^: 2957, 2925, 2875, 2856, 1741, 1462, 1368, 1274, 1181, 1083, 808, 723. ^13^C NMR (CDCl_3_) *δ*: 173.8, 170.1, 74.74, 73.55, 64.25, 63.64, 63.56, 34.25, 34.22, 31.75, 31.98, 29.62, 29.48, 29.46, 29.43, 29.37, 29.34, 29.31, 29.29, 29.28, 29.24, 29.17, 29.11, 29.03, 28.97, 28.94, 28.58, 27.56, 27.10, 26.60, 26.47, 26.16, 25.86, 25.85, 25,23, 25.01, 24.91, 24.84, 24.58, 22.70, 22.56, 21.46, 14.05, 9.01, 8.05; ^1^H NMR (CDCl_3_) *δ*: 5.0 m (2H), 4.0 *t* (*J* = 6.7 Hz, 2H), 3.89 m (2H), 2.28 *t* (*J* = 7.5 Hz, 2H), 2.07 s (5H), 1.65 m(12H), 1.50 bm (3H), 1.25 bs (56H), 0.85 *t* (*J* = 6.6, 7.1H, 6H); ^13^C CDCl_3_*δ*: 173.8, 170.4, 166.2, 75.0, 64.29, 63.61, 34.28, 34.26, 31.77, 31.12, 30.68, 29.64, 29.57, 29.55, 29.51, 29.48, 29.46, 29.40, 29.37, 29.37, 29.33, 29.32, 29. 31, 29.26, 29.19, 29.14, 29.13, 29.06, 28.99, 28.96, 28.61, 26.51, 25.88, 25.87, 25.86, 25.09, 24.93, 22.59, 22.05, 20.84, 14.02.

#### 2.3.4. Valeroylation of Tetrahydroxy Jojoba Oil

Tetrahydroxy jojoba oil (77.0 g, 125 mmol), valeric anhydride (97%, 100 g, 0.537 mol), and *p*-TsOH (0.84 g) were placed in a dry 500 mL round-bottomed flask equipped as in (a) above and heated to gentle reflux as for the acetate. Heating was stopped when a worked up sample of the reaction mixture showed no (OH) stretching mode of the starting tetrahydroxy substrate in the FT-IR spectrum. The cooled reaction mixture was poured into a beaker containing a 40/60 mixture of Na_2_CO_3_/NaCl saturated solutions. Ethyl acetate was added to facilitate separation of the phases. This mixture was washed for the third time with saturated bicarbonate to decompose and remove residual unreacted valeric anhydride. The organic phase was dried over anhydrous MgSO_4_ and concentrated to give 93.0 g (95%); d^23°C^ 1.012 g/mL. FT-IR *ν*_NaCl_ cm^−1^: 2961, 2926, 2878, 2855,1824, 1741, 1469, 1381, 1242, 1171, 1111, 1037, 937, 723; ^1^H NMR (CDCl_3_) *δ*: 5.0 bs (2H), 4.05 *t* (*J* = 6.7, 6.8 Hz, 2H), 3.9 m (2H), 2.43 *t* (*J* = 7.4, 7.5 Hz, 3H), 2.34 *t* (*J* = 7.5 Hz, 4H), 2.25 *t* (*J* = 7.4, 7.5 Hz, 3H), 1.65 m (19H), 1.52 bm (3H), 1.30 m (61H), 0.90 *t* (*J* = 7.4 Hz, 10H), 0.85 *t* (*J* = 6.7, 6.4 Hz, 6H); ^13^C NMR (CDCl_3_) *δ*: 173.80, 173.22, 173.17, 169.48, 74.69, 73.47, 64.30, 63.67, 34.92, 34.29, 34.06, 31.79, 31.12, 30.72, 29.66, 29.59, 29.57, 29.53, 29.50, 29.48, 29.42, 29.39, 29.36, 29.32, 29.31, 29.29, 29.21, 29.17, 2915, 29.13, 29.08, 29.01, 28.99, 28.63, 27.09, 26.52, 26.22, 25.89, 25.28, 25.06, 24.95, 22.60, 22.24, 22.22, 21.96, 14.03, 13.64, 13.58.

#### 2.3.5. Dynamic Rheological Property Measurements

Linear viscoelastic properties were conducted with a strain-controlled Rheometric ARES-LSM rheometer (TA Instruments, Piscataway, NJ). A torsion rectangular geometry was used to study thermomechanical properties of the tetrahydroxy jojoba wax sample. The strain sweep experiments were conducted at 1 rad/s in a strain range of 0.02–20% at −20°C to 80°C, respectively, to identify the linear range of the material. An applied shear strain valued in the linear range was adopted for the other viscoelastic property measurements. Linear viscoelasticity indicates that the measured parameters are independent of the applied shear strain. Small-amplitude oscillatory shear experiments were conducted over a frequency (*ω*) range yielding the shear storage (G′) and loss (G″) moduli. The storage modulus represents the nondissipative component of mechanical properties. Elastic or “rubber-like” behavior is suggested if the G′ spectrum is independent of frequency and greater than the loss modulus over a certain range of frequencies. The loss modulus represents the dissipative component of the mechanical properties and is a characteristic of viscous flow. The phase shift or phase angle (*δ*) is defined by *δ* = tan^−1^(G″/G′) and indicates whether a material is solid with perfect elasticity (*δ* = 0°), or liquid with pure viscosity (*δ* = 90°), or something in between (0 < *δ* < 90°). The temperature ramp measurements were conducted in a range of −20°C to 80°C with the heating rate of 2°C/min at 1 rad/s frequency and 0.05% strain. The dynamic frequency sweep experiments were carried out at 25°C and 70°C, respectively, at 0.05% strain in a frequency range of 0.1 to 100 rad/s.

## 3. Results and Discussion

**Figure 2:**
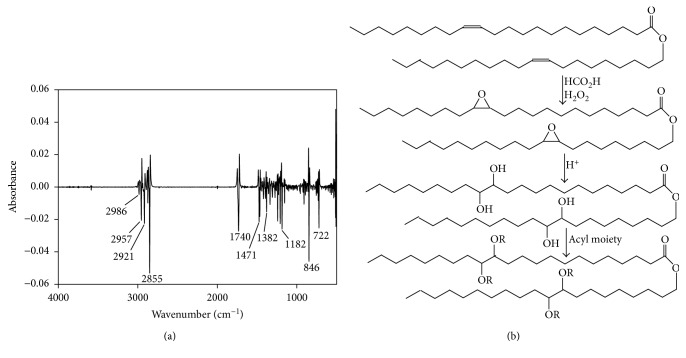
(a) 2nd derivative spectrum of diepoxy jojoba oil intermediate. (b) Epoxidation, ring opening, and acylation of tetrahydroxy jojoba intermediate to the acyl ester derivatives. R = Ac, propanoyl, pentanoyl.

**Figure 3:**
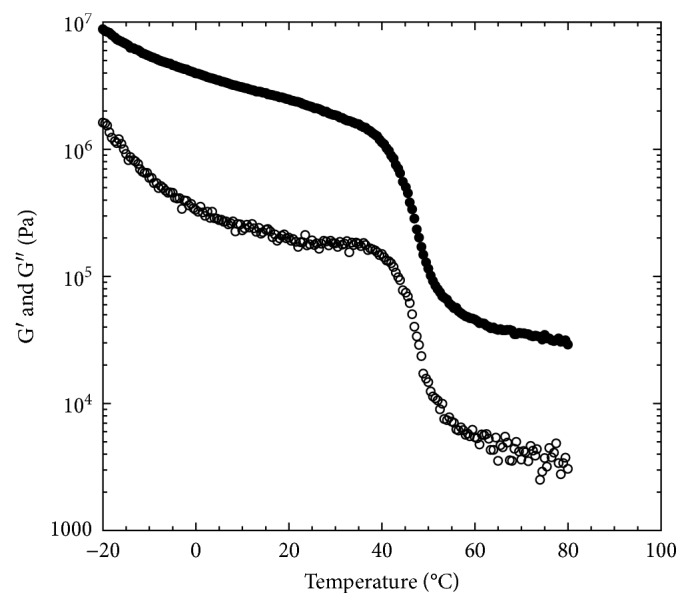
The linear viscoelastic properties of aged polyhydroxy jojoba wax with temperature sweep measurement in a range of −20°C to 80°C with the heating rate of 2°C/min at 1 rad/s frequency and 0.05% strain. Filled symbols: G′; opened symbols: G″.

The ^1^H NMR and ^13^C NMR spectra of the unmodified Purcell jojoba oil are given in Figures 1 and 1 as comparatives to those of the derivatives. These spectra of the oil are in the main first-order spectra. The four olefinic protons (H-13, H-14 and H-31, H-32) are an overlapping multiplet at 5.35 ppm, whereas the oxygenated methylene (H-23) is a downfield triplet at 4.05 ppm. The methylene group proximal to the acyl carbonyl is an upfield triplet (*J* = 8.2, 7.5 Hz, 2H) at about 2.30 ppm relative to H-23; whereas the methylene protons (H-12, H-15, H-30 and H-33) form another overlapping multiplet at 2.02 ppm; H-3 and H-24 set also overlap at 1.60 ppm with the rest of the methylene protons resonating as a broad, intense envelop at 1.30 ppm. The terminal methyl protons are observed as a triplet (*J* = 6.6, 7.1 Hz, 6H) at 0.89 ppm. The corresponding ^13^C spectrum (Figure 1) affirmed the proton spectrum. Hydration of the jojoba oil to the tetrahydroxy jojoba intermediate was via the oxirane which was carried out in a one-pot reaction. The oxirane was characterized from a sampled aliquot by its FT-IR spectrum having an additional relatively strong singlet asymmetric absorption band at 847 cm^−1^ and a H–C–O–C–H stretch at 2985 cm^−1^ (Figure 2). This 847 cm^−1^ band of the diepoxy jojoba oil is in contrast to epoxy triglycerides which consistently give an asymmetric doublet at 825–845 cm^−1^. Also, in contradistinction to triglycerides, jojoba oxirane showed a rather broad ^1^H singlet at 2.87 ppm for protons in the three-membered rings and a strong single line for the corresponding ring ^13^C spectrum at 57.12 ppm in contrast to the multiple lines in the 57 to 53 ppm spectral region in triglycerides [26, 27]. At room temperature, both the oxirane and tetrahydroxylated jojoba intermediates are colorless low-melting solids. The tetrahydroxy material gave a characteristically broad (OH) absorption band centered at 3438 cm^−1^ in its mid FT-IR spectrum. The other diagnostic FT-IR bands of the hydroxylated oil include the 2962, 2929 cm^−1^ asymmetric stretch of the –CH_3_ and –CH_2−_, respectively, and the 2874 and 2855 cm^−1^ symmetric stretching modes for the –CH_3_, –CH_2−_, respectively. A strong ester carbonyl band is observed at 1742 cm^−1^, a –CH_2_– deformation at 1470 cm^−1^ and the –CH_3_ deformation at 1374 cm^−1^. The oxirane bands at 847 cm^−1^ and 2985 cm^−1^ are absent in the tetrahydroxy material. The ^1^H NMR spectrum of the tetrahydroxy jojoba oil was characterized by the presence of additional protons, multiplets at 3.85, 3.58, and 1.78 ppm, integrating to 2H, 2H, and 4H, respectively (Figure 3). Three clean triplets are anticipated in the proton spectrum as also observed in the unmodified oil. The most downfield of these is at 4.02 ppm *t* (*J* = 6.8 Hz, 2H) assigned to H-23 (Figure 1). The second triplet is observed at 2.25 ppm *t* (*J* = 7.5 Hz, 2H) assignable to the methylene protons H-2, whereas the overlapping terminal methyl groups are observed at 0.85 ppm *t* (*J* = 6.6, 7.1 Hz, 6H) slightly upfield from those of the unmodified starting jojoba oil which resonate at 0.90 ppm. The other observed resonances include the multiplet at 3.90 ppm for the methine protons and the multiplets of the methylene protons in the rest of the long chain: 1.79 ppm m (4H), 1.52 ppm m (10H), 1.40 ppm bm (5H) ,and at 1.25 ppm bs (50H). Some observed behavioral characteristics of the tetrahydroxy jojoba oil are the following: when freshly synthesized, it crystalizes to a white solid on cooling to room temperature, but this solid readily melts on warming to 35–40˚C. Stored at room temperature for a long time period, it cures to a brittle waxy solid with rubbery characteristics. In the latter condition, the tetrahydroxy jojoba wax fails to melt even at 200°C when decomposition seems to set in from the emitted odor. In this rubbery condition, the solid has a tendency toward low solubility in organic solvents. This behavior could be attributable mainly to intra- or intermolecular hydrogen bonding in the hydroxylated structure. An attempt to acylate a long-sitting waxy sample of the tetrahydroxy material was only partially successful on account of its resistance to solubilization even in the hot anhydride. On the other hand, acylation of the freshly prepared tetrahydroxy jojoba intermediate to the tetraacylates was essentially quantitative using the respective anhydrides catalyzed by *p*-TsOH. The derivatized tetraacylates are light red to light yellow clear liquids at room temperature with densities slightly > 1.00 g/mL around room temperature. The FT-IR, ^1^H, and ^13^C spectral characteristics of these derivatives are summarized in Tables 1 and 2.  Table 1 shows the FT-IR absorbance data of the starting oil through the tetrapentanoate. In the tetraacetate, the methyl and methylene asymmetric stretching bands are observed at 2959 and 2925 cm^−1^, respectively, whereas the symmetric absorption bands are observed at 2874 and 2855 cm^−1^, respectively. The ^1^H and ^13^C NMR spectra of this product on one hand show resonances at 5.0 ppm for the methine protons; a triplet at 4.02 ppm (*J* = 6.7 Hz, 2H) is assigned to the oxygen-bearing methylene group of the fatty alcohol moiety. The resonance at 3.79 ppm, multiplet (2H) is assignable to the pair of oxygen-bearing methine groups on each segment of the oxidized molecule, while the triplet at 2.26 ppm (*J* = 7.5 Hz, 2H) is assigned to the methylene group proximal to the acyl carbon. The methyl protons of the acetoxy substituent are a strong singlet observed at 2.07 ppm. This resonance accounts for 6H of the 12H in each molecule of the derivative most probably due to symmetry reasons. The terminal methyl protons are observed at 0.85 ppm *t* (*J* = 6.7 Hz, 6H), and the corresponding terminal methyl carbons are observed at 14.04 ppm, whereas the ^13^C resonance at 20.86 ppm is assigned to the acetate substituent methyl groups (Table 2). In the tetrapentanoate compounds, the methine protons resonate at 5.05 ppm as a multiplet (2H), while its most downfield methylene protons as in the acetoxy compounds are those of the esterified fatty alcohol contiguous to the parent ester carboxyl oxygen and are observed at 4.05 ppm, *t* (*J* = 6.7, 6.8 Hz, 2H) compared to 4.02 ppm in the tetraacetate.

### 3.1. Rheology

**Figure 4:**
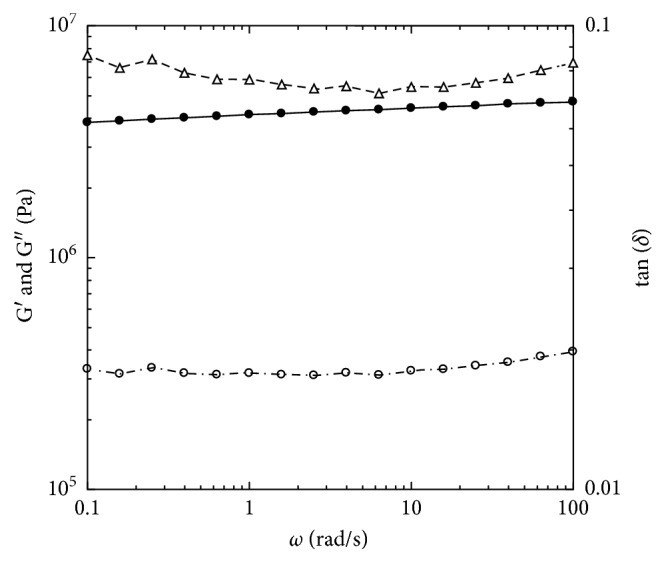
Linear viscoelastic properties of small-amplitude oscillatory shear frequency sweep experiment for the aged at 25°C with 0.05% strain. •: G′; ○: G″; ▵: tan (*δ*).

**Figure 5:**
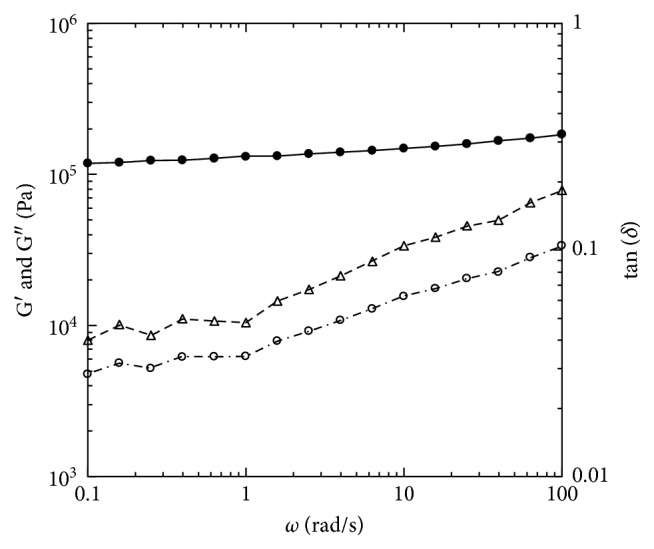
Linear viscoelastic properties of small-amplitude oscillatory shear frequency sweep experiment for the aged tetrahydroxy jojoba wax at 70°C with 0.05% strain. •: G′; ○: G″; ▵: tan (*δ*).

The linear viscoelastic behavior of aged tetrahydroxy jojoba wax with the temperature sweep measurements is shown in Figure 3. The storage modulus was higher than the loss modulus in the entire measured temperature range from −20°C to 80°C. Both moduli had a sharp decrease in the temperature range of 35°C to 55°C, which indicated the material behavior changing from a hard brittle state to a relatively rubbery state–glass transition. The glass transition temperature was 47.8 ± 1.7°C. To explore the linear viscoelastic properties of this material at both hard brittle state and the rubbery state, dynamic small-amplitude oscillatory shear experiments were conducted at lower temperature (25°C) and at higher (70°C) than the glass transition temperature. At 25°C, which was lower than the glass transition temperature, the tetrahydroxy jojoba wax exhibited strong viscoelastic solid behavior (Figure 4). Curves of both the storage and loss moduli were nearly independent of measured frequencies and were parallel. The storage modulus at 1.0 rad/s was 4.1 × 10^6^ Pa (Figure 4). The phase shifts were almost constant, in a range of 4.1° to 4.9°, within the measured frequency range, indicating pretty brittle behavior. The G′ of highly cross-linked rubber is around 10^7^ Pa, and the phase shift is around 11° at room temperature [28]. Therefore, the aged tetrahydroxy jojoba wax showed similar storage modulus as rubber but a much smaller phase shift at 25°C. This result suggested that the aged tetrahydroxy jojoba wax should have similar elasticity as the rubber-like material but exhibits much more brittle property than rubber at temperatures lower than its glass transition. At 70°C, which was higher than its glass transition temperature, the tetrahydroxy jojoba wax still exhibited viscoelastic solid behavior (Figure 5). The storage moduli (G′) were greater than the loss moduli (G″) within all measured frequencies. The curve of the storage modulus was still frequency independent over the three measured frequency decades. The values of G′ at 70°C were much lower than those at 25°C (Figures 4 and 5). The storage modulus at 1 rad/s was 1.3 × 10^5^ Pa at 70°C. The curve of the loss modulus was dependent on frequency, and the phase shifts had a wider range of 2.3° to 10.4°, which indicated that the tetrahydroxy jojoba wax exhibited a little more viscous behavior at 70°C than at 25°C. This result suggested that at temperatures higher than the glass transition, the tetrahydroxy jojoba wax should show more fluid-like behavior than at temperatures lower than its glass transition.

**Figure 6:**
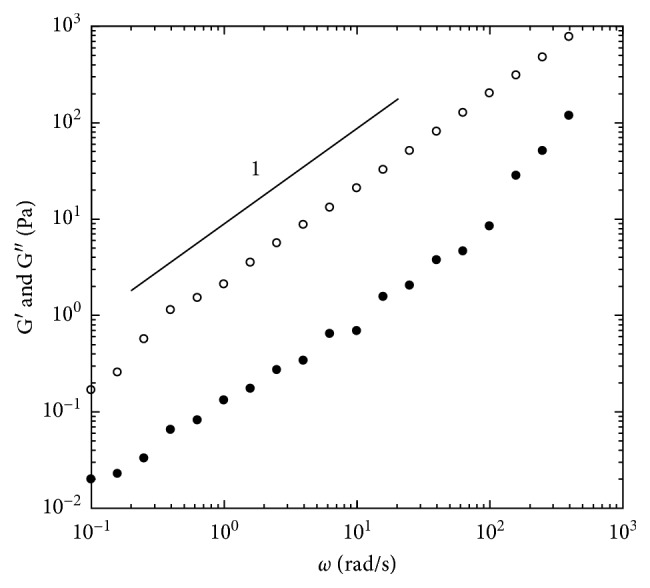
Linear viscoelastic properties of small-amplitude oscillatory shear frequency sweep experiment for the jojoba tetraacetate sample at 25°C with 1.0% strain. •: G′; ○: G″.

**Figure 7:**
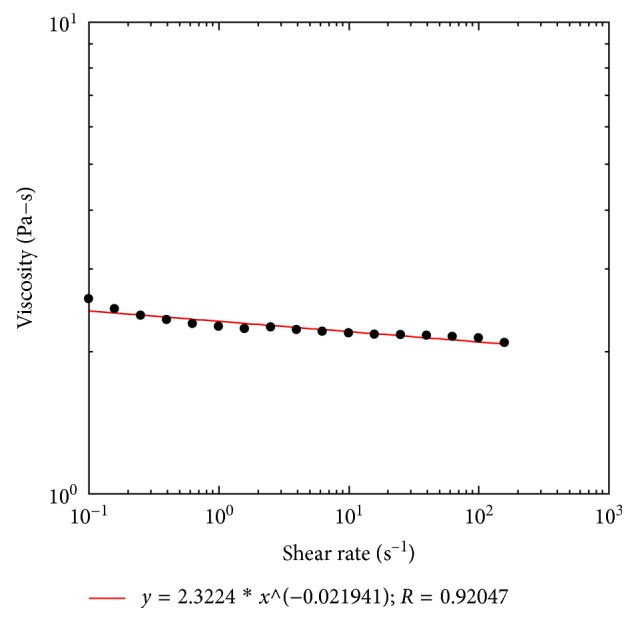
The nonlinear viscoelastic properties of the steady shear measurements for the jojoba tetraacetate sample at the temperature of 25°C. Symbols are experiment results; the line is fitted with power law model.

For the tetraacetate derivative of jojoba wax, the linear dynamic frequency sweep measurements at 25^o^C are shown in Figure 6. Both curves of storage modulus (G′) and loss modulus (G″) were very frequency dependent; and G″ was higher than G′ within the measured frequency range (Figure 6). The loss moduli (G″) followed a nearly straight line with a slope of one, which indicated that the sample exhibited viscoelastic fluid properties and was very close to that of a viscous fluid behavior [29]. The G′ at 1 rad/s for the tetraacetate was 0.13 Pa at 25^o^C, indicating very small elasticity (Figure 6). The phase shifts for the sample were in the range of 81.4^o^ to 88.1^o^, which suggests the sample to be a fluid-like material. The nonlinear steady shear viscoelastic property and a power law constitutive equation fitting the tetraacetate sample are shown in Figure 7. The sample exhibited a behavior very close to a Newtonian fluid and very slight shear-thinning (Figure 7). From the fitting data, we can see that the sample possesses power law exponent (*n*) of 0.98, which is nearly the same as the Newtonian fluid behavior (*n* = 1). The nonlinear steady shear viscoelastic property and a power law constitutive equation fitting for this sample are shown in Figure 7. The sample exhibited a behavior very close to the Newtonian fluid and very slight shear-thinning (Figure 7). From the fitting data, we can see that the jojoba tetraacetate sample possesses a power law exponent (*n*) value of 0.98, which is nearly the same as the Newtonian fluid behavior (*n* = 1).

### 3.2. Physical and Tribological Evaluations

As explained above, jojoba was chemically modified by converting its double bonds into ester branches of varying chain length. Understanding the effect of such modifications on tribological and other properties is of immense importance. In light of this, jojoba and its derivatives were evaluated for their physical and tribological properties using a variety of methods. These evaluations were conducted on the neat oils as well as on blends of the neat oils in biobased and petroleum-based base oils. The blend investigations are intended to evaluate the potential of jojoba and its derivatives to be used as additives in lubricant formulations. The neat and blend investigations are discussed separately.

### 3.3. Neat Oil Properties

Table 1 compares the physical properties of the jojoba oil with its acetate, propionate, and valerate derivatives. The first property investigated is solubility. Ample solubility is essential to allow the material to be used in blends with biobased and petroleum-based base oils as well as an additive in lubricant formulations. As shown in Table 1, chemical modification of the jojoba oil did not adversely affect solubility in the biobased high-oleic sunflower oil (HOSuO) or in the petroleum-based polyalpha olefin (PAO-6) base oils. The only exception is the acetate derivative, which displayed low solubility in PAO-6. This can be attributed to the vast difference in the polarity between PAO-6 and the acetate derivative. The acetate, because of its short chain, results in a more polar oil than the jojoba oil or the other two ester derivatives. Since PAO-6 contains no functional group, it is very nonpolar and is incompatible with the acetate derivative, which is the most polar of the four oils investigated.

Jojoba esters displayed higher density than jojoba by at least 0.1 g/mL. The density of jojoba esters increases with decreasing ester chain length, which corresponds with increasing polarity. The effect can be attributed to increased intermolecular (polar) interaction with decreasing chain length, resulting in reduced specific volume.

Chemical modification of the jojoba oil also resulted in higher kinematic viscosity (1.3 to 20-fold relative to unmodified material) that varied with the ester chain length. The observed kinematic viscosity increased in the following order: jojoba oil < jojoba propionate < jojoba valerate < jojoba acetate. Increase in viscosity can be attributed to increases in polarity and molecular weight as a result of the chemical modification. It appears that polarity might be the predominant factor for the acetate derivative, whereas molecular weight might be the predominant factor for the valerate derivatives.

In general, chemical modification resulted in reduced viscosity index (VI) of all the jojoba esters. The reduction was most severe for the acetate derivative, which gave a value that was less than half of that for the unmodified jojoba oil (Table 1). The reduction was less severe for the other two derivatives. The observed VI values varied with substituent chain structure and increased in the following order: jojoba acetate < jojoba valerate < jojoba propionate < jojoba.

The effect of chemical modification on cold flow properties is summarized in Table 2. The data show good improvement in cloud point and an excellent improvement in pour point due to esterification. Thus, relative to the jojoba oil, the esters displayed a reduction of 10–17°C in the cloud point and a reduction of 56–63°C in the pour point. Even though the introduction of side chain was expected to improve flow properties by suppressing the tendency of the oil to crystalize, such a large effect on the pour point was not predictable. Such low pour point values are usually observed for selected synthetic petroleum-based base oils, such as PAOs, and very few biobased synthetic oils without pour point depressant additives [30]. Thus, these ester modifications help overcome one of the major drawbacks of biobased base oils related to poor cold flow properties without the use of pour point depressants.

Table 2 also compares the onset (OT) and peak (PT) oxidation temperatures of the jojoba ester derivatives relative to the unmodified jojoba oil. As expected, the conversion of the double bonds in the jojoba oil to the esters resulted in improved oxidation stability as shown by the increase in OT and PT values of the modified oils. The modification of jojoba removes reactive allylic and bis allylic protons which causes oxidation reactions to occur. As a result, OT and PT values increased by ≥ 15°C, which is a significant improvement and helps overcome the second major drawback of biobased oils related to oxidative stability. Thus, the conversion of the jojoba oil to the corresponding esters results in biobased base oils with the highly improved pour point and oxidation stability.

Tribological properties of the neat jojoba oil and its ester derivatives are compared in Table 3. The evaluation was conducted on a 4-ball tribometer under antiwear (AW, ASTM D-4172) and extreme pressure (EP, ASTM D-2783) conditions. The antiwear investigation showed that the coefficient of friction (COF) of the jojoba esters was lower than that of the unmodified jojoba oil and varied with the ester structure. The COF increased with increasing ester chain length as follows: jojoba acetate < jojoba propionate < jojoba valerate < unmodified jojoba oil. The wear data, which are expressed in terms of wear scar diameter (WSD), showed that the esters have poorer wear property than the unmodified jojoba oil, which also varied with ester chain length. Thus, the WSD of the modified esters decreased with increasing ester chain length in the following order: jojoba acetate > jojoba propionate > jojoba valerate > unmodified jojoba oil.

The EP investigation showed a slight improvement in EP properties due to the chemical modification of jojoba to esters. Thus, the weld point of the jojoba oil increased from 120 kgf before chemical modification to 140–160 kgf for the esters (Table 3). The slight improvement in weld point can be attributed to the slightly higher molecular weight of the esters. Such low weld point results are expected since jojoba oil or its esters contain none of the EP active elements (e.g., S, P, and Cl) in their structures [31].

### 3.4. Blend Properties

Jojoba oil and its ester derivatives were also investigated for their additive properties in PAO-6. Of particular interest is the effect of these biobased materials on the viscosity and viscosity index (VI) of PAO-6. Blends with up to 10% (w/w) of jojoba and its derivatives in PAO-6 were investigated. The resulting data are compared to the values for the neat oils in Tables 4–7.

Blending did not result in any dramatic changes in viscosity or VI (Tables 4–7). In general, the blends displayed properties that were intermediate between the neat materials, with values consistent with the blend composition and predictable with simple mixing rules. Thus, addition of jojoba resulted in gradual increases in density and VI and gradual decreases in viscosity to the value for the neat jojoba oil (Table 4). Similar results were observed with blends containing jojoba acetate (Table 5), jojoba propionate (Table 6), and jojoba valerate (Table 7). Closer examination of Tables 4–7 shows some minor exceptions to the general trend discussed above. For example, blend of PAO-6 with 1% w/w jojoba-acetate resulted in higher VI than those for the neat components (Table 5). Blends of PAO-6 with up to 10% w/w jojoba propionate displayed lower viscosity than the neat materials (Table 6). The result of this investigation shows that jojoba oil and its ester derivatives have very limited potential as viscosity index improver.

## Figures and Tables

**Table 1:** Room temperature solubility and kinematic/dynamic viscosity and viscosity index (VI) of the neat jojoba oil and ester derivatives.

Property	ASTM method	Jojoba oil	Jojoba esters		
			Acetate	Propionate	Valerate
RT sol (% w/w)	Visual	—	—	—	—
PAO-6	—	>50	<3.2	>50	>50
OSuO	—	>50	>50	>50	>50
Density (g/cm^3^)	D-7042	—	—	—	—
40°C	—	0.8487 ± 0.0002	0.9617 ± 0.0000	0.9618 ± 0.0002	0.9435 ± 0.0001
100°C	—	0.8101 ± 0.0000	0.9189 ± 0.0001	0.9161 ± 0.0000	0.9011 ± 0.0000
kVisc (mm^2^/s)	D-7042	—	—	—	—
40°C	—	21.8 ± 0.0	176.2 ± 0.2	39.9 ± 0.0	76.8 ± 0.2
100°C	—	5.9 ± 0.0	18.5 ± 0.1	7.8 ± 0.0	12.5 ± 0.0
VI	D-2270	242	117	170	161

**Table 2:** Cold flow and oxidation stability (by PDSC method) of the neat jojoba oil and ester derivatives.

Property	ASTM method	Jojoba oil	Jojoba esters		
			Acetate	Propionate	Valerate
Cloud point (°C)	D-2500	9 ± 0.0	−2.0 ± 0.0	−1.0 ± 0.0	−8.0 ± 0.0
Pour point (°C)	D-97	9.0 ± 0.0	−47.0 ± 1.7	−51.0 ± 0.0	−54.0 ± 0.0
PDSC-OT (°C)	D-6186	176.50 ± 0.49	192.99 ± 0.53	193.57 ± 0.30	196.47 ± 1.15
PDSC-PT (°C)	D-6186	193.84 ± 0.45	208.99 ± 0.16	208.90 ± 1.32	210.73 ± 1.05

**Table 3:** Four-ball antiwear and extreme pressure properties of the neat jojoba oil and ester derivatives.

Property	ASTM method	Jojoba oil	Jojoba esters		
			Acetate	Propionate	Valerate
4-ball AW	D-4172	—	—	—	—
	D-5183				
COF	—	0.065 ± 0.001	0.046 ± .000	0.044 ± 0.003	0.050 ± 0.002
wsd (mm)	—	0.589 ± 0.014	0.811 ± 0.042	0.756 ± 0.029	0.634 ± 0.030
4-ball EP	D-2783	—	—	—	—
weld point (kgf)	—	120	160	140	140

**Table 4:** Effect of [jojoba] on PAO-6 blend density, viscosity, and VI.

	[Jojoba], % w/w				
	0 (PAO-6)	1	5	10	100 (jojoba)
Density, g/cm^3^					
40°C	0.8112 ± 0.0002	0.8115 ± 0.0001	0.8130 ± 0.0001	0.8150 ± 0.0001	0.8487 ± 0.0002
75°C	0.7894 ± 0.0001	0.7898 ± 0.0001	0.7912 ± 0.0000	0.7931 ± 0.0000	0.8261 ± 0.0001
100°C	0.7739 ± 0.0000	0.7743 ± 0.0000	0.7757 ± 0.0000	0.7775 ± 0.0000	0.8101 ± 0.0000
kVisc, mm^2^/s					
40°C	30.4 ± 0.0	30.2 ± 0.0	29.5 ± 0.0	28.7 ± 0.0	21.8 ± 0.0
75°C	10.2 ± 0.0	10.2 ± 0.0	10.1 ± 0.0	10.0 ± 0.0	9.3 ± 0.0
100°C	5.8 ± 0.0	5.8 ± 0.0	5.8 ± 0.0	5.8 ± 0.0	5.9 ± 0.0
dVisc, mPa·s					
40°C	24.7 ± 0.0	24.51 ± 0.01	24.0 ± 0.0	23.41 ± 0.00	18.46 ± 0.00
75°C	8.0 ± 0.0	8.03 ± 0.01	8.0 ± 0.0	7.90 ± 0.00	7.66 ± 0.03
100°C	4.5 ± 0.0	4.52 ± 0.00	4.5 ± 0.0	4.51 ± 0.00	4.80 ± 0.01
VI	136	140	144	150	242

**Table 5:** Effect of [jojoba-acetate] on PAO-6 blend density, viscosity, and VI.

	[Jojoba-acetate], % w/w		
	0 (PAO-6)	1	100 (jo-ac)
Density, g/cm3			
40°C	0.8112 ± 0.0002	0.8127 ± 0.0000	0.9617 ± 0.0000
75°C	0.7894 ± 0.0001	0.7908 ± 0.0001	0.9367 ± 0.0000
100°C	0.7739 ± 0.0000	0.7753 ± 0.0000	0.9189 ± 0.0001
kVisc, mm^2^/s			
40°C	30.4 ± 0.0	30.8 ± 0.0	176.2 ± 0.2
75°C	10.2 ± 0.0	10.3 ± 0.0	39.1 ± 0.1
100°C	5.8 ± 0.0	5.9 ± 0.0	18.5 ± 0.1
dVisc, mPa·s			
40°C	24.7 ± 0.0	25.0 ± 0.0	169.5 ± 0.2
75°C	8.0 ± 0.0	8.2 ± 0.0	36.6 ± 0.1
100°C	4.5 ± 0.0	4.6 ± 0.0	17.0 ± 0.0
VI	136	139	117

**Table 6:** Effect of [jojoba-propionate] on PAO-6 blend density, viscosity, and VI.

	[Jojoba-propionate], % w/w				
	0 (PAO-6)	1	5	10	100 (jo-pr)
Density, g/cm3					
40°C	0.8112 ± 0.0002	0.8125 ± 0.0001	0.8172 ± 0.0001	0.8235 ± 0.0000	0.9618 ± 0.0002
75°C	0.7894 ± 0.0001	0.7907 ± 0.0001	0.7952 ± 0.0000	0.8013 ± 0.0001	0.9351 ± 0.0000
100°C	0.7739 ± 0.0000	0.7752 ± 0.0000	0.7795 ± 0.0000	0.7854 ± 0.0000	0.9161 ± 0.0000
kVisc, mm2/s					
40°C	30.4 ± 0.0	30.3 ± 0.0	30.0 ± 0.05	29.70 ± 0.0	39.9 ± 0.0
75°C	10.2 ± 0.0	10.2 ± 0.0	10.14 ± 0.04	10.1 ± 0.0	13.5 ± 0.0
100°C	5.8 ± 0.0	5.8 ± 0.0	5.83 ± 0.01	5.8 ± 0.0	7.8 ± 0.0
dVisc, mPa·s					
40°C	24.7 ± 0.0	24.6 ± 0.0	24.5 ± 0.0	24.5 ± 0.0	38.4 ± 0.0
75°C	8.0 ± 0.0	8.1 ± 0.0	8.1 ± 0.0	8.1 ± 0.0	12.6 ± 0.0
100°C	4.5 ± 0.0	4.5 ± 0.0	4.5 ± 0.0	4.6 ± 0.0	7.2 ± 0.0
VI	136	140	142	144	170

**Table 7:** Effect of [jojoba-valerate] on PAO-6 blend density, viscosity, and VI.

	[Jojoba-valerate], % w/w				
	0 (PAO-6)	1	5	10	100 (jo-val)
Density, g/cm3					
40°C	0.8112 ± 0.0002	0.8122 ± 0.0001	0.8167 ± 0.0000	0.8224 ± 0.0001	0.9435 ± 0.0001
75°C	0.7894 ± 0.0001	0.7904 ± 0.0000	0.7948 ± 0.0000	0.8003 ± 0.0000	0.918 7± 0.0000
100°C	0.7739 ± 0.0000	0.7749 ± 0.0000	0.7792 ± 0.0000	0.7846 ± 0.0000	0.9011 ± 0.0000
kVisc, mm2/s					
40°C	30.4 ± 0.0	30.6 ± 0.0	31.2 ± 0.0	32.1 ± 0.0	76.8 ± 0.2
75°C	10.2 ± 0.0	10.2 ± 0.0	10.4 ± 0.0	10.8 ± 0.0	23.1 ± 0.0
100°C	5.8 ± 0.0	5.9 ± 0.0	6.0 ± 0.0	6.2 ± 0.0	12.5 ± 0.0
dVisc, mPa·s					
40°C	24.7 ± 0.0	24.8 ± 0.0	25.4 ± 0.0	26.4 ± 0.0	72.5 ± 0.2
75°C	8.0 ± 0.0	8.1 ± 0.0	8.3 ± 0.0	8.6 ± 0.0	21.2 ± 0.0
100°C	4.5 ± 0.0	4.6 ± 0.0	4.7 ± 0.0	4.8 ± 0.0	11.2 ± 0.0
VI	136	139	141	143	161
